# Supplementary material for: Defective lytic transglycosylase disrupts cell morphogenesis by hindering cell wall de-O-acetylation in Neisseria meningitidis
Source: eLife. 2020 Feb 5;9:e51247. doi: 10.7554/eLife.51247 (PMC7083599; doi:10.7554/eLife.51247)
Supplement: Figure 4—source data 1. [file elife-51247-fig4-data1.docx]

| **Muropeptide** | **Observed mass**  **(m+H^+^)** | **Theoretical mass**  **(m+H^+^)** | **Proportion of total muropeptides (%)** | | | | | | **Fold-change** | **Fold-change (%)** |
| --- | --- | --- | --- | --- | --- | --- | --- | --- | --- | --- |
|  |  |  | **WT** | | **(SD)** | **Helix30** | | **(SD)** | **WT /Helix30** | **WT /Helix30** |
| GM3 | 871.38 | 871.38 | 2.34 | (±0.19) | | 1.56 | (±0.16) | | 0.67 | 33**↓** |
| GM4 | 942.41 | 942.42 | 11.14 | (±0.33) | | 13.60 | (±0.47) | | 1.22 | 22**↑** |
| GM5 | 1013.45 | 1013.45 | 4.82 | (±0.05) | | 3.45 | (±0.10) | | 0.72 | 28**↓** |
| GM*3 | 913.39 | 913.39 |  |  |  |  |  |  |  |  |
| **GM*4** | **984.42** | **984.43** | **4.41** | **(±0.45)** | | **8.89** | **(±0.37)** | | **2.02** | **102↑** |
| GM4-4 | 1385.61 | 1385.62 | 2.98 | (±0.12) | | 2.96 | (±0.12) | | 0.99 | 1**↓** |
| GM*5 | 1055.46 | 1055.64 |  |  |  |  |  |  |  |  |
| GM4-GM4 | 1865.80 | 1865.81 | 10.56 | (±0.50) | | 9.54 | (±0.42) | | 0.90 | 10**↓** |
| GM4-GM5 | 1936.84 | 1936.85 | 6.25 | (±0.08) | | 3.96 | (±0.09) | | 0.63 | 37**↓** |
| **GM*4-GM4** | **1907.86** | **1907.82** | **6.73** | **(±0.59)** | | **9.35** | **(±0.23)** | | **1.39** | **39↑** |
| **GM*4-GM*4** | **1949.83** | **1949.83** | **3.24** | **(±0.37)** | | **4.73** | **(±0.21)** | | **1.46** | **46↑** |
