# Supplementary material for: Defective lytic transglycosylase disrupts cell morphogenesis by hindering cell wall de-O-acetylation in Neisseria meningitidis
Source: eLife. 2020 Feb 5;9:e51247. doi: 10.7554/eLife.51247 (PMC7083599; doi:10.7554/eLife.51247)
Supplement: Supplementary file 1. — * indicates O-acetylated MurNAc. Acetylated muropeptides are highlighted in pink. Where multiple muropeptides coeluted as a single peak, bold text indicates the most abundant mass detected. [file elife-51247-supp1.docx]

| **Peak** | **Observed mass (m+H^+^)** | **Theoretical mass (m+H^+^)** | **Structure** |
| --- | --- | --- | --- |
| 1 | 499.2123 | 499.2139 | GM |
| 2 | 462.2178 | 462.2200 | 4 |
|  |  |  |  |
| 3 | 533.2557 | 533.2571 | 5 |
| 4 | 699.2915 | 699.2936 | GM2 |
|  | 871.3758 | 871.3784 | GM3 |
| 5 | 541.2227 | 541,2245 | GM* |
| 6 | 739.3336 | 739.3362 | M4 |
|  | 942.4123 | 942.4155 | GM4 |
|  | 980.3593 |  |  |
| 7 | 1013.4517 | 1013.4526 | GM5 |
|  | 913.3880 | 913.3890 | GM3* |
|  | 1035.4336 | 1035.4370 | anhyGM5* |
| 8 | 905.4196 | 905.4216 | 4 + 4 |
| 9 | 984.4233 | 984.4261 | GM4* |
| 10 | 1385.6134 | 1385.6171 | GM4 + 4 |
| 11 | 851.3494 | 851.3522 | anhGM3 |
| 12 | 1865.8047 | 1865.8127 | GM4 + GM4 |
| 13 | 1936.8438 | 1936.8498 | GM4 + GM5 |
| 14 | 922.3881 | 922.3893 | anhyGM4 |
|  | 1427.6236 | 1427.6277 | GM4* - 4 |
|  | 1907.8177 | 1907.8232 | GM4+GM4* |
| 15 | 922.3881 | 922.3893 | anhyGM4 |
|  | 1978.8539 | 1978.8603 | GM4-GM5* |
| 16 | 1949.8286 | 1949.8338 | GM4*-GM4* |

| **Peak** | **Observed mass (*m+H^+^*)** | **Theoretical mass (*m+H^+^*)** | **Structure** |
| --- | --- | --- | --- |
| 1 | 499.21 | 499.21 | GM |
| 2 | 462.22 | 462.22 | 4 |
| 3 | 533.26 | 533.26 | 5 |
| 4 | 699.29 | 699.29 | GM2 |
|  | **871.38** | **871.38** | **GM3** |
| 5 | 541.22 | 541.22 | GM* |
| 6 | 739.33 | 739.34 | M4 |
|  | **942.41** | **942.42** | **GM4** |
| 7 | 1013.45 | 1013.45 | GM5 |
|  | **913.39** | **913.39** | **GM*3** |
|  | **1035.43** | **1035.44** | **GanhyM*5** |
| 8 | 905.42 | 905.42 | 4 - 4 |
| 9 | 984.42 | 984.43 | GM*4 |
| 10 | 1385.61 | 1385.62 | GM4 - 4 |
| 11 | 851.35 | 851.35 | GanhyM3 |
| 12 | 1865.80 | 1865.81 | GM4 - GM4 |
| 13 | 1936.84 | 1936.85 | GM4 - GM5 |
| 14 | **1427.62** | **1427.63** | **GM*4 - 4** |
|  | 1907.82 | 1907.82 | GM4 - GM*4 |
| 15 | **922.39** | **922.39** | **GanhyM4** |
|  | 1978.85 | 1978.86 | GM4 - GM5* |
| 16 | 1949.83 | 1949.83 | GM*4 - GM*4 |

Table X. Muropeptides identified by mass spectrometry. Numbers correspond to HPLC chromatogram peaks annotated in figure X. G, GlcNAc ; M, MurNAc ; anhyM, anhydroMurNAc ; 3, L-Ala – D-Glu – mDAP ; 4, L-Ala – D-Glu – mDAP – D-Ala ; 5, L-Ala – D-Glu – mDAP – D-Ala – D-Ala ; * indicates *O*-acetylated MurNAc. Acetylated muropeptides are highlighted pink. Where multiple muropeptides co-eluted as a single peak, bold text denotes the most abundant mass detected.
