# Supplementary material for: Defective lytic transglycosylase disrupts cell morphogenesis by hindering cell wall de-O-acetylation in Neisseria meningitidis
Source: eLife. 2020 Feb 5;9:e51247. doi: 10.7554/eLife.51247 (PMC7083599; doi:10.7554/eLife.51247)
Supplement: Supplementary file 2. [file elife-51247-supp2.docx]

| **Table 1** Data-collection and [refinement statistics](http://reference.iucr.org/dictionary/Statistics) | | | | | |
| --- | --- | --- | --- | --- | --- |
| Values in parentheses are for the outer shell. | | | | | |
| **Data Collection** | **LtgA** |  |  |  |  |
|  |  |  |  |  |  |
| Data collection | | | | | |
| Wavelength (Å) | 0.9795 |  |  |  |  |
| Resolution range (Å) | 49.84-1.98 (2.067-1.995) |  |  |  |  |
| Space group | *P2_1_2_1_2_1_* |  |  |  |  |
| Unit-cell parameters | | | | | |
| *a* (Å) | 66.82 |  |  |  |  |
| *b* (Å) | 72.25 |  |  |  |  |
| *c* (Å) | 122.31 |  |  |  |  |
| 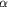 (°),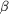 (°),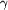 (°) | 90 |  |  |  |  |
|  |  |  |  |  |  |
|  |  |  |  |  |  |
| Total reflections | 299848 |  |  |  |  |
| Unique reflections | 40499 |  |  |  |  |
| Multiplicity | 7.4 (7.4) |  |  |  |  |
| Completeness (%) | 98.63 (94.17) |  |  |  |  |
| Mean *I*/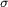(*I*) | 16.40 (1.47) |  |  |  |  |
| Wilson *B* factor (Å^2^) | 43.22 |  |  |  |  |
| *R*_merge_^[#](http://onlinelibrary.wiley.com/iucr/doi/10.1107/S1399004714000911/mn5048bdy.html#TFN1)^ | 0.055 (0.063) |  |  |  |  |
| Refinement | | | | | |
| *R* factor[^+^](http://onlinelibrary.wiley.com/iucr/doi/10.1107/S1399004714000911/mn5048bdy.html#TFN2) | 0.1929(0.3192) |  |  |  |  |
| *R*_free_ | 0.2464(0.3645) |  |  |  |  |
| No. of atoms | 4661 |  |  |  |  |
| No. of waters | 271 |  |  |  |  |
| No. of protein residues | 563 |  |  |  |  |
| R.m.s.d., bonds (Å) | 0.007 |  |  |  |  |
| R.m.s.d., angles (°) | 0.96 |  |  |  |  |
| Ramachandran favored (%) | 98 |  |  |  |  |
| Ramachandran outliers (%) | 0 |  |  |  |  |
| *B* factors (Å^2^) | | | | | |
| Average | 51.00 |  |  |  |  |
| Macromolecules | 50.80 |  |  |  |  |
| Ligand  Solvent  All-atom clash score | -------  54.60  2.09 |  |  |  |  |
|  |  |  |  |  |  |
|  | | | | | |

^†^*R*_merge_ = Σ_hkl_Σ_i_∣*I*_i_(*hkl*) −〈*I* _(_*_hkl_*_)_〉∣/Σ_hkl_Σ_I_ *I* _(_*_hkl_*_)_

^‡^*R*_factor_ = Σ_hkl_ |∣*F* _obs_ − *F* _calc_∣|/Σ_hkl_ |*F* _obs_|

**R*_free_ was computed identically except that all reflections belonged to a test set consisting of a 10% random selection of the data.
